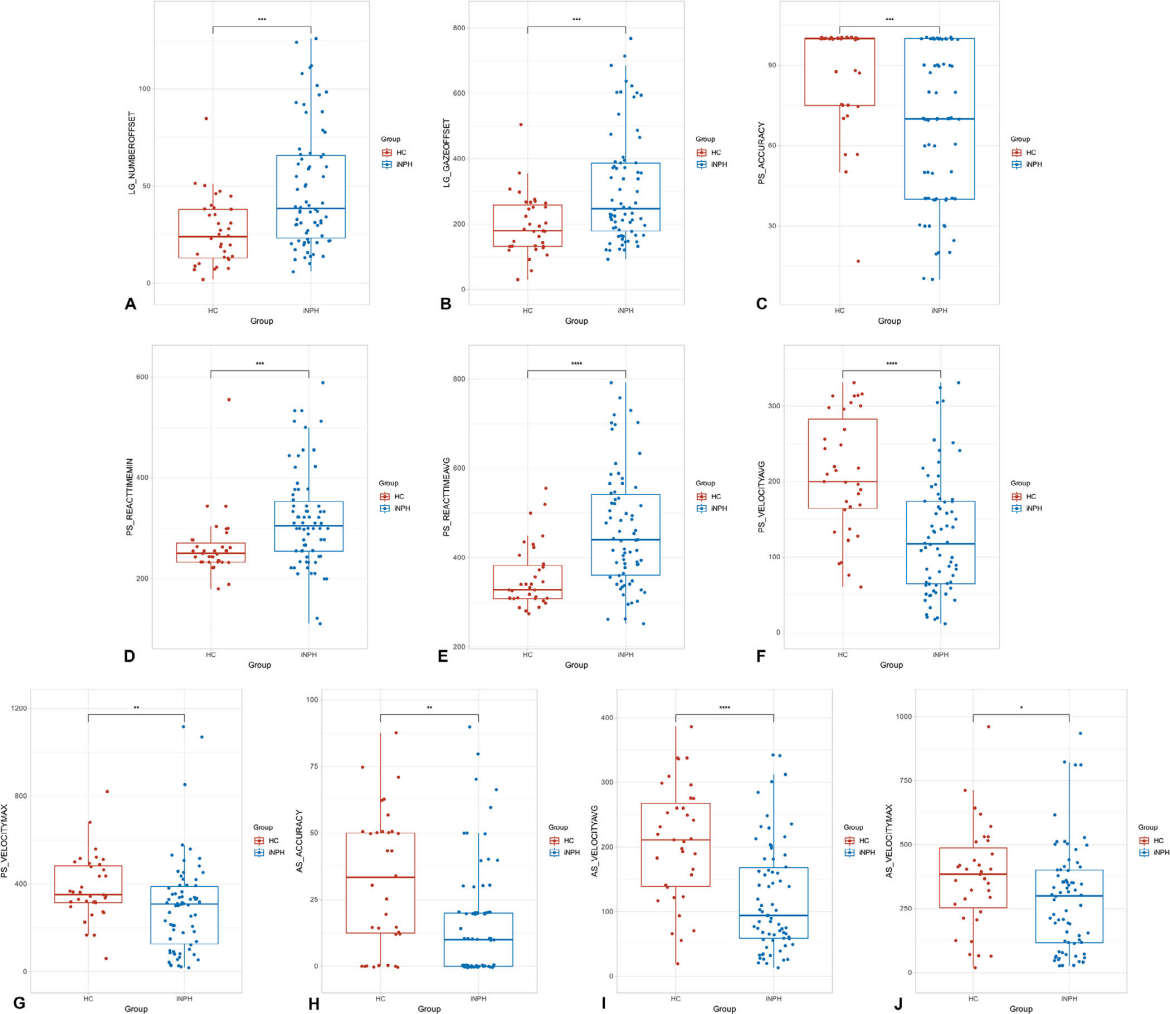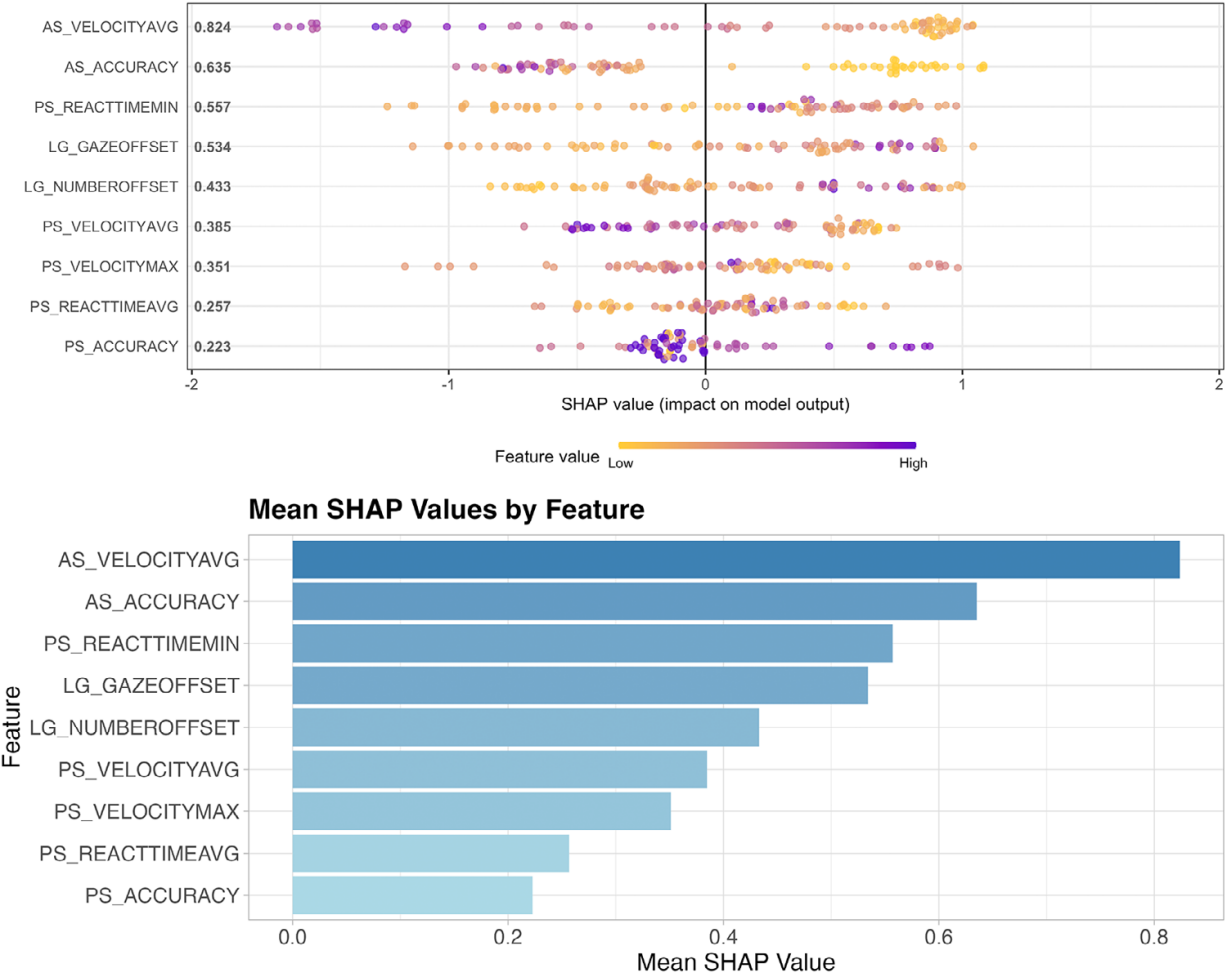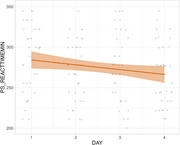# Eye‐tracking assessments in the diagnosis of patients with idiopathic normal pressure hydrocephalus

**DOI:** 10.1002/alz70856_098953

**Published:** 2025-12-24

**Authors:** Tianlu Ma, Hanlin Cai, Keru Huang, Feng Yang, Hui Gao, Shiyu Feng, Linyuan Qin, Ruihan Wang, Shan Wang, Qian Liao, Na Hu, Yi Liu, Jiaojiang He, Liangxue Zhou, Qin Chen

**Affiliations:** ^1^ West China Hospital of Sichuan University, Chengdu, Sichuan, China

## Abstract

**Background:**

Eye‐tracking assessments can reflect early cognitive impairment in patients with neurodegenerative diseases. The diagnosis of idiopathic normal pressure hydrocephalus (iNPH) is challenging due to unspecific symptoms and insensitive diagnostic methods. The diagnostic value of eye‐tracking assessments is unclear in patients with iNPH. This study aimed to investigate the eye movement abnormalities of patients with iNPH and the role of eye‐tracking assessment in the diagnosis of iNPH during continuous external lumbar drainage (ELD).

**Method:**

Patients diagnosed with possible iNPH (*n* = 70) and age‐, sex‐, and education‐matched healthy controls (*n* = 35) were enrolled in an ongoing prospective cohort study at the West China Hospital of Sichuan University from May 2022 to February 2024. All participants completed baseline eye‐tracking assessments, including fixation, pro‐saccade, and anti‐saccade tasks. Patients with iNPH also underwent continuous ELD and daily cognitive, gait tests and eye tracking assessments. XGBoost model was used for differentiating iNPH from healthy controls and general estimated equation was used to analyze daily changes of eye movement metrics during ELD.

**Result:**

Patients with iNPH exhibited significantly lower accuracy (*p* <0.001), slower velocity (*p* <0.001), and longer reaction time (*p* <0.001) in eye tracking tasks compared to healthy controls. The XGBoost model obtained excellent diagnostic efficacy for differentiating between iNPH and healthy controls (AUC = 0.89). For patients who received continuous external lumbar drainage, continuous improvement was found in the pro‐saccade task's minimal reaction time during the period of ELD (*p* = 0.022).

**Conclusion:**

Our study demonstrates that patients with iNPH exhibited lower accuracy, velocity, and longer reaction time during eye‐tracking assessment. Moreover, eye‐tracking assessment offers an objective and quantitative methods for diagnosing iNPH and monitoring ELD responses.